# Awake Craniotomy for Resection of Diffuse Astrocytoma During Pregnancy: A Case Report

**DOI:** 10.7759/cureus.39016

**Published:** 2023-05-14

**Authors:** Sabina Oblitas López, Jhon E Bocanegra-Becerra, Nicole M Castillo-Huerta, Alonso Ludeña-Esquivel, Alicia Becerra Zegarra

**Affiliations:** 1 Department of Neurosurgery, Hospital Edgardo Rebagliati Martins, Lima, PER; 2 Neurosurgery Simulation and Innovation Lab, Mayo Clinic, Arizona, USA; 3 School of Medicine Alberto Hurtado, Universidad Peruana Cayetano Heredia, Lima, PER

**Keywords:** case report, pregnancy, glioma, diffuse astrocytoma, brain tumor, awake craniotomy

## Abstract

Brain tumors rarely present during pregnancy; however, a life-threatening interaction may develop between maternal and disease factors. Moreover, awake surgery has been an infrequent treatment option during this life stage. We contribute to this knowledge gap by presenting the case of a 33-year-old woman who developed tonic-clonic seizures during the 18th week of pregnancy due to a neoplastic lesion near the left motor area. A multidisciplinary team performed an awake craniotomy for tumor resection and the histopathological examination revealed a diffuse astrocytoma. On the follow-up, radiotherapy was administered and the patient delivered a healthy newborn at week 37.

## Introduction

Newly diagnosed brain tumors rarely present during pregnancy. A frequency of 3-6 per 10^6^ live births have been reported [1]. Although intracranial tumors can rarely complicate the course of pregnancy, they may severely affect the well-being of the mother and fetus [2]. Moreover, the physiological changes associated with pregnancy (e.g., hormonal changes and increased maternal blood flow) could exacerbate the mother's symptoms, alter the natural history of the disease, and detriment the fetus’s vitality [3].

Gliomas have been the most frequently reported primary brain tumors in this stage with few cases treated with awake surgery [4]. Awake craniotomies are well-established and preferred for preserving patients' neurologic status with lesions within or adjacent to eloquent brain areas; however, there are scarce reports on the feasibility of tumor resection through awake craniotomy in pregnant women [5]. We contribute to the paucity of evidence by presenting the case of a patient who underwent awake resection of a brain tumor during pregnancy. Importantly, we highlight the multidisciplinary approach that led to an optimized postoperative course and the delivery of a healthy newborn during the follow-up. Informed consent was obtained for this study.

## Case presentation

**Figure 1:**
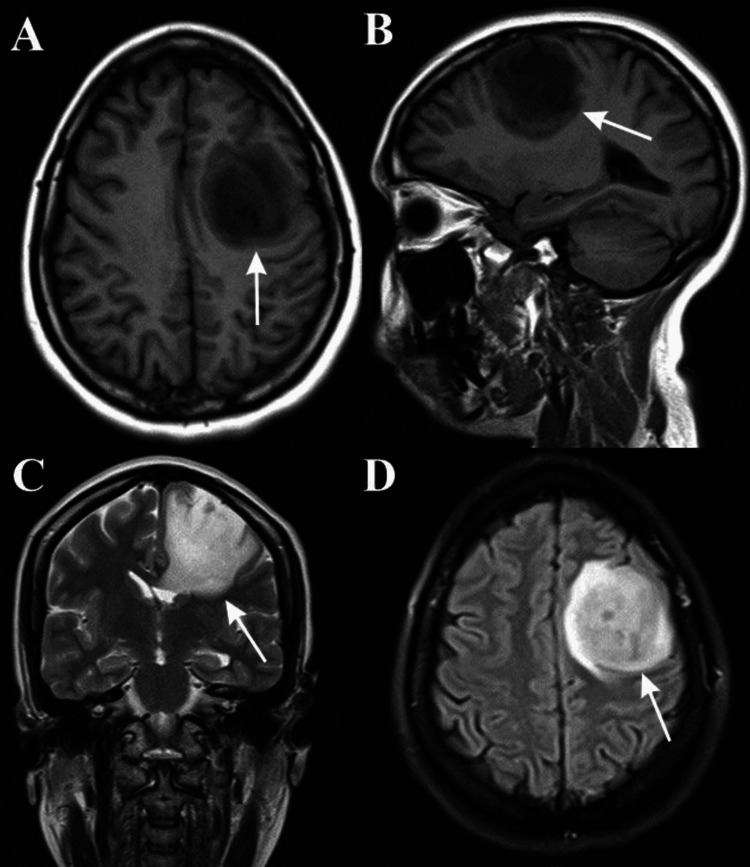
Initial brain magnetic resonance imaging Left frontal proliferative lesion of 46.9 x 43.6 x 52.6 mm causing deformation of the frontal gyrus and midline shift. The lesion was hypointense in the non-contrast axial (A) and sagittal (B) T1-weighted sequence, and hyperintense in the T2-weighted (C) and fluid-attenuated inversion recovery (FLAIR) (D) sequences.

A 33-year-old woman in the 18th week of pregnancy presented with a history of two episodes of tonic-clonic seizures and severe headaches that worsened progressively. The neurologic examination revealed right hemiparesis and a brain magnetic resonance imaging (MRI) showed a left frontal proliferative lesion causing deformation of the frontal gyrus which was hypointense in the T1-weighted sequence and hyperintense in the T2-weighted and fluid-attenuated inversion recovery (FLAIR) sequences (Figure 1). The lesion showed no contrast enhancement and no diffusion restriction.

A collaborative effort was established between the departments of Neurosurgery, Obstetrics and Gynecology, Psychology, and Anesthesiology. Because the patient remained symptomatic and with a high risk of further cerebral herniation, the best treatment decision was to perform an awake craniotomy with intraoperative fetal monitoring. The patient was educated about the risks and benefits of surgery and the maneuvers she would need to perform to map the language and sensorimotor functions intraoperatively.

Surgery

**Figure 2:**
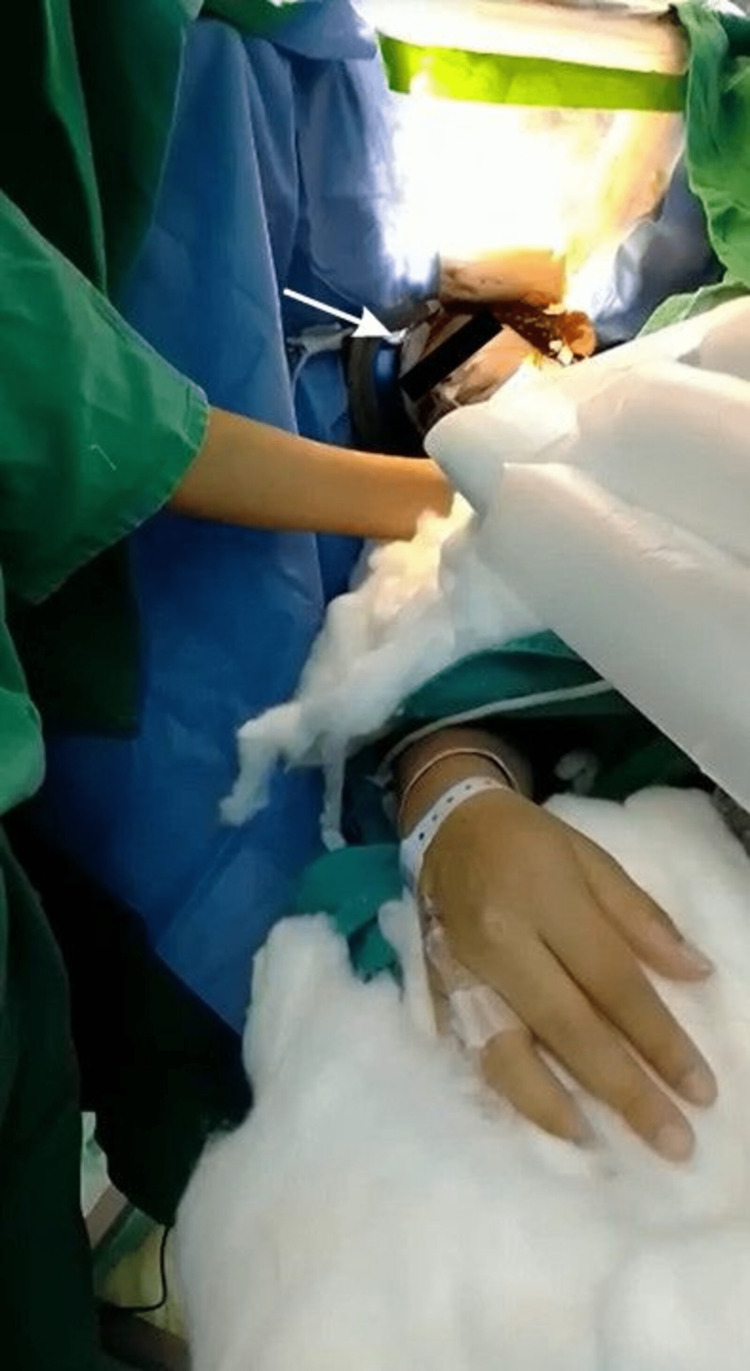
Intraoperative neurological examination with the patient awake

The patient was placed in a right lateral decubitus position for brain tumor excision surgery (Figure 2). The anesthesia team performed scalp block using xylocaine and bupivacaine mixed 1:1. A pterional craniotomy was performed under conscious sedation administering an intravenous infusion of remifentanil and propofol for anesthetic and intravenous acetaminophen and remifentanil for analgesia. The fetal status was monitored via ultrasound by the Obstetrics and Gynecology team before and after the surgery. They were also prepared in case an emergent delivery would be needed. Intraoperatively, a whitish and vascularized lesion was located at the left middle frontal gyrus. Stimulation of the cerebral cortex confirmed the tumor location anterior to the primary motor cortex without its involvement. Besides, the patient's neurologic function was tested by asking her to follow commands such as grasping the finger of the assisting surgeons, counting from one to ten, sticking out the tongue, and moving the hand's fingers. Stimulation parameters were 3-4 mAmp of 60 Hz and positive polarity. During the piecemeal resection, careful subcortical stimulation using an Ojemann stimulator with a bipolar tip was performed to demarcate the lesion borders and avoid damage to the motor and language areas. A fresh frozen section was sent for histopathological examination, which revealed a diffuse astrocytoma, classified as World Health Organization (WHO) grade II glioma. The postoperative course was uneventful. The patient had a moderate headache postoperatively managed with intravenous acetaminophen, and her neurological examination was normal without any motor or sensory deficits.

Follow-up

**Figure 3:**
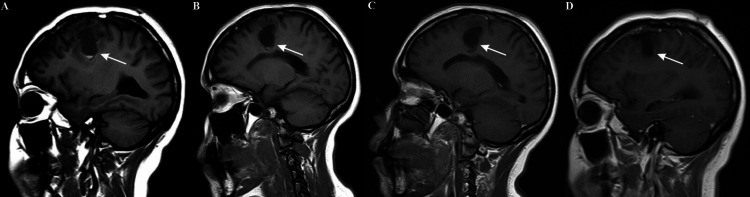
Brain magnetic resonance imaging during the follow-up Sagittal view of T1-weighted sequences with contrast at two months (A), three months (B), four months (C), and nine months (D).

A two-month follow-up MRI showed an 8 mm residual tumor piece next to a cystic non-tumoral component (Figure 3A), which remained invariable in size in a three-month follow-up brain MRI (Figure 3B). The patient was scheduled for an elective c-section on week 37th (four months after surgery) and delivered a healthy newborn. A four-month follow-up brain MRI revealed the patency of the residual mass (Figure 3C) and because of the development of seizures, the patient received radiotherapy 66Gy over 33 fractions in two months. A nine-month follow-up brain MRI showed a small cortical contrast-enhancing residual tumor of less than 10 mm plus a porencephalic cyst of 32 x 24 x 22 mm hypointense in the T1-weighted sequence associated with post-radiation changes in the cerebral parenchyma (Figure 3D). Currently, the patient remains asymptomatic and takes levetiracetam 1g PO q12hr.

## Discussion

Brain tumors are infrequent during pregnancy, with gliomas accounting for the majority of cases followed by meningiomas and acoustic neuromas [2, 3]. A complex interaction can develop as a result of the physiological changes associated with pregnancy and tumor-related factors. For example, the increased maternal blood flow can predispose to the aggravation of the secondary symptoms of tumor growth and raised intracranial pressure, which further difficult the clinical diagnosis because of the resemblance to non-tumoral diseases during pregnancy [6]. Moreover, the hormonal changes from pregnancy such as the production of progesterone, vascular endothelial growth factor, and placental growth factor might play a role in tumor growth [7-9].

Because of these changes during pregnancy, the clinical presentation of tumors is usually exacerbated, which may precipitate obstetrical emergencies [8, 10]. Signs and symptoms are related to increased intracranial pressure and alteration of the cortical brain signaling such as headache, nausea, vomiting, seizures, and neurological deficits. Other symptoms can include gait disturbance, urinary incontinence, and memory loss [2, 10]. Computed tomography (CT) and magnetic resonance imaging (MRI) constitute available diagnostic images with particular indications during pregnancy. The easier acquisition of a CT scan makes it a convenient option for diagnosis in the acute setting. On the other hand, a brain MRI, considered safe by the American College of Obstetricians and Gynecologists (ACOG), provides a better resolution for the delineation of the tumor [5]. It is worth mentioning that the use of gadolinium should be discouraged unless strictly necessary since there is no conclusive evidence in humans, with animal models showing teratogenic potential [9, 11].

Treatment modalities during this life stage include medical management, radiotherapy, and surgery. Medical management refers to the use of chemotherapy and corticosteroids to alleviate cerebral edema, with the additional benefit of maturing the fetus’ lungs [9, 8]. Surgery has been infrequently reported, with the majority of cases performed under general anesthesia because a cerebrospinal fluid (CSF) leak might cause significant neurologic morbidity in presence of an intracranial mass lesion [12]. Notably, the surgical decision relies on the presenting symptoms, patient clinical stability, and gestational age [5]. For instance, during the first and second trimesters, gestational advancement and delayed surgical resection can be advised for patients in stable conditions, with radiotherapy as a second-line option. Nevertheless, if patients are clinically unstable (e.g., sudden, severe neurological conditions, or intracranial hypertension) or present worsening deterioration of neurological symptoms during follow-up, such as in the case of our patient, immediate surgery becomes the mandatory option [2, 5, 13]. In addition, if a malignant glioma is suspected, tumor removal should be performed without delay, regardless of gestational age, as there is a risk of invasion of the cerebral parenchyma [13].

Awake surgery allows optimization of the maximal extent of tumor resection in eloquent areas while the surgeon monitors the neurological function in real-time; thus, reducing postoperative deficits [14]. Usually, the second trimester of pregnancy is the ideal time window and warrants careful multidisciplinary planning between Neurosurgery, Gynecology and Obstetrics, Psychology, and Anesthesiology [2]. As a result, the collaboration among specialties allows for detailed patient education, intraoperative fetal monitoring, and appropriate neurological evaluation in the operating room. Of particular significance, the anesthesiology team must consider the preoperative psychological and technical preparation of the patient, and also the various potential intraoperative complications associated with awake craniotomy (e.g., difficult emergency intubation, seizures, hemorrhage, intracranial hypertension, and agitation) [3, 15]. In the scientific literature, few reports have illustrated the use of this technique, with most of the cases using a combined regimen of anesthetics such as remifentanil, alfentanil, dexmedetomidine, and propofol [5, 16]. The latter is preferred over volatile agents in healthy pregnant patients, and it has the advantage of being less disruptive to motor-evoked potential recording during awake surgery [16, 17]. Even though among anesthetic agents, none of these have been demonstrated to have teratogenic effects when using standard concentrations at any gestational age [11], they have the risk of maternal respiratory depression, which should be closely monitored. Finally, fetal monitoring should be evaluated during surgery, taking into account changes induced by anesthesia. Even though there is no solid evidence about the intraoperative use of fetal heart rate (FHR) monitoring and fetal outcomes in non-obstetric surgery, it is recommended to individualize assessment based on gestational age, type of surgery, and available facilities [18].

Overall, awake craniotomy during pregnancy is a reasonable treatment option for select patients with a stable gestational course and lesions compromising eloquent brain areas.

## Conclusions

This case study provides an example of the feasibility of performing an awake craniotomy on a patient in the second trimester of pregnancy with a brain tumor and deteriorating neurological function. In addition, we emphasized the importance of a multidisciplinary treatment approach to optimize maternal and fetal clinical outcomes. The main limitations are the fact that this is a single case and that some specific tests could not be performed due to the low-income patient's context. Given the paucity of literature regarding the safety and long-term outcomes of this approach in pregnant patients, further large-scale studies are necessary to elucidate its potential risks and benefits. Thus, such efforts could serve to refine clinical management strategies and improve the patient prognosis of the mother and fetus.
